# An annotated list of the Chamaemyiidae (Diptera, Acalyptrata) of Turkey with new records and additional data

**DOI:** 10.3897/zookeys.838.33027

**Published:** 2019-04-11

**Authors:** Martin J. Ebejer, Miroslav Barták

**Affiliations:** 1 Entomology Section, Department of Natural Sciences, National Museum of Wales, Cathays Park, Cardiff, UK Department of Natural Sciences, National Museum of Wales Cardiff United Kingdom; 2 Department of Zoology and Fisheries, Faculty of Agrobiology, Food and Natural Resources, Czech University of Life Sciences Prague, Kamýcká 129, 165 00 Praha-Suchdol, Czech Republic Czech University of Life Sciences Prague Praha Czech Republic

**Keywords:** Silver-flies, faunistics, taxonomy, distribution

## Abstract

A list of all the species of Chamaemyiidae known from Turkey is compiled from the literature and supplemented by new records. A total of 40 species in five genera is given with updated nomenclature. One undescribed species is illustrated but not named for lack of males. The distribution of each species outside Turkey is summarised.

## Introduction

Silver flies of the family Chamaemyiidae have an interesting biology with a potential for the biological control of pest species of aphids and adelgids (Aphidoidea) and scales and mealybugs (Coccoidea) that attack crops, horticultural plants, and forest trees. Silver flies are found in all continents except Antarctica, but much remains to be discovered in nearly all zoogeographical regions.

When compared to the other zoogeographical regions, the Palaearctic is relatively well studied with many species having been associated with their prey and the prey with their host plants, largely summarised by Tanasijtshuk (1986). Nevertheless, the distribution of most species remains inadequately known and there is always more to discover about the biology of a majority of the species. Southern Europe and Turkey through to Central Asia is a region rich in species, reflecting the diversity of habitats and flora. No doubt, more species await discovery and description. Their taxonomy can be difficult owing to the very similar external appearance of species within each genus.

The first record of a species of Chamaemyiidae from Turkey appears to be that of Süreyya and Hovasse (1931). They record larvae of *Leucopis* sp. very successfully attacking the scale insect *Marchalinahellenica* Genn. damaging pine trees on Princes Islands (Turkey). Bodenheimer (1953) reported on an unspecified species of *Leucopis* Meigen, 1830. Three species of *Neoleucopis* McAlpine, 1971 were examined by McAlpine in his revision of the genus (McAlpine 1971). Several more species were added by various authors since then (Eichhorn 1968; McAlpine 1978; Düzgüneş et al. 1982, Tanasijtshuk 1986, Elmali 1997; Kaydan et al. 2006, Raspi and Ebejer 2008, Raspi 2013). In the last two articles, the authors added more data and in each publication a new species of *Parochthiphila* Czerny, 1904 was described. Kaydan et al. (2006) also gave the prey species and their host plants. In a recent paper, Satar et al. (2015) gave a summary of the biological and ecological role of species in this family, added four new records for Turkey and provided biological data based from their own rearing records of several species of *Leucopis*. They gave no new records of species in other genera.

The aim of this article is to briefly review what is known of the Turkish fauna based on the literature, recent field work of one of us (MB) and supplementary material collected by Dr Jindřich Roháček (Opava, Czech Republic). We list all the species recorded in these earlier papers and add new records for the country and further locality and chorological data on some previously known species. Nomenclature is updated.

## Materials and methods

Species are listed in alphabetical order under each genus. Previous records are cited below each species name. Additional locality data based on the recently collected material is included and new records for Turkey are indicated. Depositories of specimens are in the M Barták collection, Czech University of Life Sciences, Prague, unless otherwise stated and given in parenthesis at the end of each data entry thus: **MJE** – MJ Ebejer collection, Cowbridge, UK; **MSO** – Museum Silesiae Opava, Czech Republic. Material cited in this paper was collected by water pan traps (PT), Malaise traps (MT), and by hand held sweep net (SW).

The material treated here originates mainly from Muğla province (Muğla, Akyaka, Toparlar, Gökçeova Gölü, and Dalyan), and some from Samsun province (Samsun). The general distribution of species is summarised mainly from Tanasijtshuk (1984, 1986), Beschovski (1995), Beschovski and Merz (1998), Ebejer (2017), and Raspi (2013). Taxonomy follows Tanasijtshuk (1986) and Raspi and Benelli (2016).

## List of species

**CHAMAEMYIINAE**

***Chamaemyia* Meigen, 1803**

***Chamaemyiaaridella* (Fallén, 1823)**

Raspi and Ebejer 2008: 61

**Distribution**: widespread in Europe, from Britain south to the Mediterranean and Turkey.

***Chamaemyiaemiliae* Tanasijtshuk, 1970**

**Material examined**: 2♂♂, Muğla, 700 m, university campus, MT, 37°09'42"N, 28°22'21"E, 17–22.v.2011; 1♀, 12 km SW of Muğla, 660 m, on *Ferulacommunis*, 37°07'40"N, 28°16'28"E, 23.v.2011; 1♂, Muğla, 720 m, university campus, MT, 37°09'42"N, 28°22'13"E, xi.2015–iv.2016, H Pala leg.

**Distribution**: Hungary and Russia eastwards to Kazakhstan. New record for Turkey.

***Chamaemyiageniculata* (Zetterstedt, 1838)**

**Material examined**: 1♀, Antalya, Yarpuz, 4.7 km W nr cross-road, 1240 m, 37°07'26"N, 31°48'01"E, 16.v.2011, J Roháček leg. (MSO); 1♂, Antalya, Ürünlü, 5.8 km SW, Manavgat River, 440 m, 37°04'30"N, 31°39'25"E, 17.v.2011, J Roháček leg. (MSO).

**Distribution**: A widespread species in Europe through Ukraine to Middle Asian states and Mongolia. New record for Turkey.

***Chamaemyiajuncorum* (Fallén, 1823)**

**Material examined**: 1♂, Gökçeova Gölü, lake shore, 1750 m, 37°03'42.52"N, 28°48'28.42"E, 20.ix.2012; 1♂, Akyaka, 30 m, forest, SW, 37°03'19"N, 28°19'36"E, 30.iv.–9.v.2013.

**Distribution**: Widespread across the whole Palaearctic including North Africa. New record for Turkey.

***Chamaemyiapolystigma* (Meigen, 1830)**

Raspi 2013: 24

**Material examined**: 1♀, Antalya, Ödaönü, 1 km S, Alara River shores, 11–13 m, 36°40'24"N, 31°40'57"E, 13.v.2011, J Roháček (MSO); 1♂3♀♀, Antalya, Murtiçi, 1 km S, 490–510 m, 36°52'20"N, 31°46'03"E, 31°40'57"E, 14.v.2011, J Roháček (MSO); 1♀, Antalya, Emiraşıklar, 1 km NW, 950 m, 37°02'45"N, 31°43'48"E, 17.v.2011, J Roháček (MSO); 1♀, Antalya, Ibradı, 3.7 km NW, 1200 m, 37°07'15"N, 31°34'10"E, 17.v.2011, J Roháček (MSO); 1♀, Akyaka, river bank, salty meadow, 37°03'16"N, 28°19'57"E, 16–27.v.2011; 1♀, 11 km E of Muğla, wood + meadow, 1310 m, 37°12'45"N, 28°27'42"E, 1.v.2013; 1♂1♀, Samsun, university campus, 41°22'N, 36°11'E, 22.vi–4.vii.2014; 1♀, Akyaka, 40 m, forest, SW, 37°03'16"N, 28°19'35"E, 26.iv.2016; 1♀, Toparlar, lowland forest, 8 m, SW+PT, 36°59'27"N, 28°38'50"E, 28–30.iv.2016.

**Distribution**: Widespread in Europe and North Africa, Turkey, and reaches Mongolia.

***Chamaemyiasylvatica* Collin, 1966**

**Material examined**: 1♂1♀, Muğla, 710 m, university campus, MT, 37°09'39"N, 28°22'20"E, xi–iii.2013; 1♂, 11 km E of Muğla, wood + meadow, 1310 m, 37°12'45"N, 28°27'42"E, 1.v.2013; 2♂♂, 13 km NE of Muğla, pinewood + pasture, 1100–1300 m, 37°15'N, 28°30'E, 2–3.v.2016.

**Distribution**: Britain and Central Europe to Poland and Bulgaria. New record for Turkey.

***Parochthiphila* Czerny, 1904**

**Parochthiphila (Parochthiphila) inconstans (Becker, 1903)**

**Material examined**: 1♂, Muğla Province, Köyceğiz, Toparlar, waterfall, 44 m, 36°49'N, 28°58'E, 26.iv.2006; 1♂, Muğla, 730 m, university campus, MT, 37°09'38"N, 28°22'11"E, 5–19.viii.2015, H Kavak leg.

**Distribution**: Iberian Peninsula, Mediterranean islands, North Africa and Arabia. New record for Turkey.

**Parochthiphila (Parochthiphila) spectabilis (Loew, 1858)**

Raspi and Ebejer 2008: 61

**Material examined**: 3♂♂4♀♀, Antalya, Manavgat, 4.4 km S, Manavgat rivershore, 1 m, 36°45'01"N, 31°28'03"E, 15.v.2011, J Roháček leg. (MSO); 1♀, Antalya, Manavgat, 3.5 km S, Titreyen lake, 1 m, 36°45'25"N, 31°27'19"E, 15.v.2011, J Roháček leg. (MSO); 16♂♂8♀♀, Akyaka, river bank, salty meadow, 37°03'16"N, 28°19'57"E, 16–27.v.2011; 2♂♂1♀, same data (MJE); 4♂♂, Akyaka, pasture, 4 m, 37°03'09"N, 28°20'17"E, 23–27.ix.2012; 5♂♂1♀, Toparlar, lowland wood, 60 m, 36°58'39"N, 28°39'30"E, 5–7.v.2013; 5♂♂, Akyaka, pasture, 6 m, SW, 37°03'19"N, 28°20'07"E, 28.iv.–8.v.2013; 1♂, same data (MJE); 5♂♂, Akyaka, salty meadow, SW+PT, 37°12'45"N, 28°27'42"E, 28.iv.–9.v.2013.

**Distribution**: Widespread in Europe, Turkey, through Russia to the Urals and Kazakhstan.

**Parochthiphila (Euestelia) argentiseta Ebejer & Raspi, 2008**

**Material examined**: 1♂, Samsun, university campus, 41°22'N, 36°11'E, 22.vi.–4.vii.2014; 1♂, 13 km NE of Muğla, pine wood, 1200, 37°14'50"N, 28°30'E, 23–27.vi.2015.

**Distribution**: Described and so far known only from Turkey.

**Parochthiphila (Euestelia) decipia Tanasijtshuk, 1986**

Kaydan et al. 2006: 333

**Distribution**: Italy, Moldova, Turkey, through the Middle Asian states to Afghanistan.

**Parochthiphila (Euestelia) ephesi Raspi, 2013**

Raspi, 2013: 14

**Distribution**: Described and so far known only from Turkey.

**Parochthiphila (Euestelia) frontella (Rondani, 1874)**

Raspi and Ebejer 2008: 61

**Material examined**: 2♂, Dalyan, farm, MT, 1 m, 36°48'54"N, 28°39'04"E, 8–20.viii.2015, Dursun; 1♂, Muğla, 710 m, university campus, MT, 37°09'39"N, 28°22'20"E, xi–iii.2013; 1♂, Dalyan, orchard, 4 m, 36°49'37"N, 28°39'39"E, 11.ix.2014; 2♂♂, Muğla, 720 m, university campus, 37°09'42"N, 28°22'13"E, 26–27.vi.2015; 1♂, Muğla, 730 m, university campus, MT, 37°09'38"N, 28°22'11"E, 5–19.viii.2015, H Kavak leg.

**Distribution**: Southern France, Iberia, Italy, and Mediterranean islands to Greece and the Aegean part of Turkey.

**Parochthiphila (Euestelia) kimmerica Tanasijtshuk, 1968**

Raspi and Ebejer 2008: 61

**Material examined**: 2♂♂1♀, Muğla, 700 m, university campus, SW+PT, 37°09'42"N, 28°22'21"E, 29.iv.–10.v.2011; 2♂, 12 km SW of Muğla, 660m, on *Ferulacommunis*, 37°07'40"N, 28°16'28"E, 23.v.2011; 1♂, Akyaka, 30 m, forest, SW, 37°03'16"N, 28°19'35"E, 30.iv.–9.v.2013; 1♂, Akyaka, 40 m, forest, SW, 37°03'19"N, 28°19'36"E, 26.iv.2016 .

**Distribution**: from western Russia south to Turkey and Israel.

**Parochthiphila (Euestelia) nigripes (Strobl, 1900)**

Raspi and Ebejer 2008: 61; Raspi 2013: 20

**Material examined**: 1♂, Muğla, 700 m, university campus, MT, 37°09'42"N, 28°22'21"E, 17–22.v.2011; 1♂, 11 km E of Muğla, pinewood + meadow, 1310 m, 37°12'45"N, 28°27'42"E, 23.v.2011; 1♂, 12 km SW of Muğla, 660 m, on *Ferulacommunis*, 37°07'40"N, 28°16'28"E, 23.v.2011; 1♀, Akyaka, 30 m, forest, SW, 37°03'16"N, 28°19'35"E, 30.iv.–9.v.2013; 4♂♂1♀, Muğla, 700 m, university campus, SW+PT, 37°09'42"N, 28°22'21"E, 29.iv.–10.v.2013; 2♂♂, 13 km NE of Muğla, pinewood, 1200, 37°14'50"N, 28°30'E, 23–27.vi.2015; 1♂, Muğla, 720 m, university campus, MT, 37°09'42"N, 28°22'13"E, 26–27.vi.2016.

**Distribution**: Spain through to southern Russia, Ukraine, Balkan states, Turkey, Iran, and Afghanistan.

***LEUCOPINAE***

***Leucopis* Meigen, 1830**

***Leucopisafghanica* Tanasijtshuk, 1998**

**Material examined**: 1♀, Muğla, 730m, university campus, MT, 37°09'38"N, 28°22'11"E, xi.2015–iv.2016; 1♂, Muğla, 720 m, university campus, MT, 37°09'42"N, 28°22'13"E, iv.–v.2016, H Kavak leg.; 1♀, same data, but H Pala leg.

**Distribution**: Previously known only from Afghanistan. New record for Turkey.

***Leucopisannulipes* Zetterstedt, 1848**

Düzgüneş et al. 1982: 92 (as *Leucopiscaucasica* Tanasijtshuk, 1961); Tanasijtshuk 1986: 244; Yoldaş et al. 2011: 63; Satar et al. 2015: 175

**Material examined**: 1♂, 13km NE of Muğla, pine wood + pasture, 1100–1300 m, 37°15'N, 28°30'E, 2–3.v.2016.

**Distribution**: all of Europe to western Russia, Turkey, and Iran.

***Leucopisargentata* Heeger, 1848**

*Leucopisconciliata* McAlpine & Tanasijtshuk, 1972: 1871; Düzgüneş et al. 1982: 93

Material examined: 1♂2♀♀, Antalya, Ödaönü, 1 km S, Alara River shores, 11–13 m, 36°40'24"N, 31°40'57"E, 13.v.2011, J Roháček leg. (MSO); 3♂♂1♀, Akyaka, river bank, salty meadow, 37°03'16"N, 28°19'57"E, 16–27.v.2011; 6♂♂, Akyaka, pasture, 4 m, 37°03'09"N, 28°20'17"E, 23–27.ix.2012; 10♂♂, Akyaka, salty meadow, SW+PT, 37°12'45"N, 28°27'42"E, 28.iv.–9.v.2013; 2♂♂, same data (MJE); 3♂♂, Toparlar, lowland wood, 60 m, 36°58'39"N, 28°39'30"E, 5–7.v.2013; 2♂♂, Akyaka, pasture, 8 m, 37°03'11"N, 28°20'33"E, 27.iv.2016.

**Distribution**: Central and southern Europe and from the Iberian Peninsula to Turkey and the Middle East including the Arabian Peninsula, and to Mongolia.

***Leucopisartemisiae* Tansijtshuk, 1986**

Raspi and Ebejer 2008: 62

**Distribution**: Southeastern Russia, Turkey.

***Leucopiscompacta* Tanasijtshuk, 1972**

Tanasijtshuk 1986: 269

**Distribution**: France and Bulgaria through Ukraine, Turkey, and Middle Asian states to Mongolia.

***Leucopisformosana* Hennig, 1938**

Satar et al. 2015: 175

**Distribution**: one of the most widespread species of the genus occurring from Cape Verde Islands to Cyprus and Middle East including Arabia, and in the Far East from China south through Asian countries to Australia. In tropical Africa found from Côte d’Ivoire to east, South Africa, and on the Mascarene Island of Réunion in the Indian Ocean. A full account of this species is given in Tanasijtshuk (1999).

***Leucopisgallicola* Tanasijtshuk, 1972**

Şahbaz and Uysal 2006: 122

**Distribution**: Russia, Turkey, Iran, and Middle Asian states.

***Leucopisglyphinivora* Tanasijtshuk, 1958**

Düzgüneş et al. 1982: 93; Tanasijtshuk 1986: 292; Raspi and Ebejer 2008: 63; Satar et al. 2015: 175

**Material examined**: 1♂, Antalya, Manavgat, 7 km SE, mouth of Manavgat River, 0–1 m, 36°44'17"N, 31°29'44"E, 11.v.2011, J Roháček leg. (MSO); 1♀, Antalya, Güçlüköy, 2 km E, 610 m, 36°49'06"N, 31°46'21"E, 15.v.2011, J Roháček leg. (MSO); 1♂, Muğla, 700 m, university campus, MT, 37°09'42"N, 28°22'21"E, 17–22.v.2011; 5♂♂, Akyaka, river bank, salty meadow, 37°03'16"N, 28°19'57"E, 16–27.v.2011; 1♂, Toparlar, lowland wood, 60 m, 36°58'39"N, 28°39'30"E, 5–7.v.2013; 1♂, 5 km S of Muğla, on flowers, 670 m, 37°08'27"N, 28°22'05"E, 6.v.2013.

**Distribution**: Iberian Peninsula through Europe and south to the Mediterranean and Turkey, through the Middle East to Mongolia.

***Leucopisgrunini* Tanasijtshuk, 1979**

**Material examined**: 1♀, Muğla, 700m, university campus, MT, 37°09'42"N, 28°22'21"E, 17–22.v.2011; 1♂1♀, Muğla, 700m, university campus, MT, 37°09'42"N, 28°22'21"E, iv.–v.2013, O Dursun leg.

**Distribution**: Italy, Cyprus, southern Russia, and Middle Asian states. New record for Turkey.

***Leucopishennigrata* McAlpine, 1978**

Eichhorn 1968: 210 (as *Leucopis* n. sp.); McAlpine 1978: 350

**Distribution**: Germany, France, Switzerland, Austria, former Yugoslavia, Greece, Turkey, and introduced into Canada (found in British Columbia, Alberta, New Brunswick, Newfoundland), and USA (found in Washington, Oregon, Arizona).

***Leucopiskerzhneri* Tanasijtshuk, 1970**

Elmali 1997: 174

**Distribution**: North Africa, Greece, Mongolia.

***Leucopisminuscula* Rondani, 1875**

Şahbaz and Uysal 2006: 122 (as *Leucopisauraria* Tanasijtshuk, 1961)

**Distribution**: Italy, Malta, eastern Russia, Mongolia.

***Leucopismonticola* Tanasijtshuk, 1961**

Raspi and Ebejer 2008: 63

**Distribution**: Iberian Peninsula through Central Europe to Russia, Ukraine, Turkey.

***Leucopisninae* Tanasijtshuk, 1966**

Düzgüneş et al. 1982: 94; Tanasijtshuk 1986: 272

**Material examined**: 1♂, Antalya, Manavgat, 7 km SE, Titreyen lake, 0–1 m, 36°44'17"N, 31°29'44"E, 11.v.2011, J Roháček leg. (MSO); 1♀, Antalya, Dolbazlar, 1.3 km NW, 21 m, 36°51'01"N, 31°24'24"E, 15.v.2011, J Roháček leg. (MSO); 2♂♂1♀, Akyaka, pasture, 4 m, 37°03'08.9"N, 28°20'17.4"E, 16–22.ix.2012; 1♀, Akyaka, pasture, 8 m, 37°03'11"N, 28°20'33"E, 27.iv.2016.

**Distribution**: England through Europe to southern Russia, Bulgaria, Ukraine, through the Middle East and north Africa, and to the Middle Asian states through to Mongolia.

***Leucopispallidolineata* Tanasijtshuk, 1961**

Düzgüneş et al. 1982: 94; Tanasijtshuk 1986: 257; Raspi and Ebejer 2008: 63

**Material examined**: 5♂♂, Muğla, 720 m, university campus, MT, 37°09'42"N, 28°22'13"E, 26–27.vi.2016.

**Distribution**: Central Europe through southern Russia, Ukraine, through Middle Asian states and Mongolia.

***Leucopispseudomelanopus* Tanasijtshuk, 1961**

Düzgüneş et al. 1982: 95; Tanasijtshuk 1986: 306

**Distribution**: Central Europe, southern Russia, Ukraine, Middle Asia.

***Leucopisrevisenda* Tanasijtshuk, 1970**

Satar et al. 2015: 175

**Distribution**: Central Europe, southern Russia, Ukraine, through Middle Asian states and Mongolia.

***Leucopisrufithorax* Tanasijtshuk, 1958**

Satar et al. 2015: 175

**Distribution**: Central and southern Europe, southern Russia, Ukraine, through Middle Asian states and Mongolia.

***Leucopisspyrothecae* Raspi, 2003**

Satar et al. 2015: 175

**Distribution**: Italy, Turkey.

***Leucopis* sp. n.**

Figs 1–4

**Material examined**: 1♀, Muğla, 730m, university campus, MT, 37°09'38"N, 28°22'11"E, xi.2015–iv.2016; 1♀, Muğla, 720 m, university campus, 37°09'42"N, 28°22'13"E, iv–v.2016, H Pala leg.

**Remarks**. This distinctive species appears to be undescribed, but for lack of males it cannot be named here. Another dark species of *Leucopis* (*L.albostriata* Czerny, 1936) exhibits distinct sexual dimorphism and so it may eventually prove difficult to correctly associate males with these specimens in the future. The two specimens noted here are dark, shiny, brownish black with a thin coating of pollinosity only on the head and on the pleura. Neither specimen is teneral, but one has the palp and the whole antenna yellow and the other has the palp, pedicel, and post pedicel dark brown. In other respects they are identical. Such small differences can be attributed equally to closely related species or to intraspecific variation. This supports our caution in not naming this species. The safest way to determine if these are one or two species would be to rear males and females simultaneously from a single colony.

**Figures 1–4.**
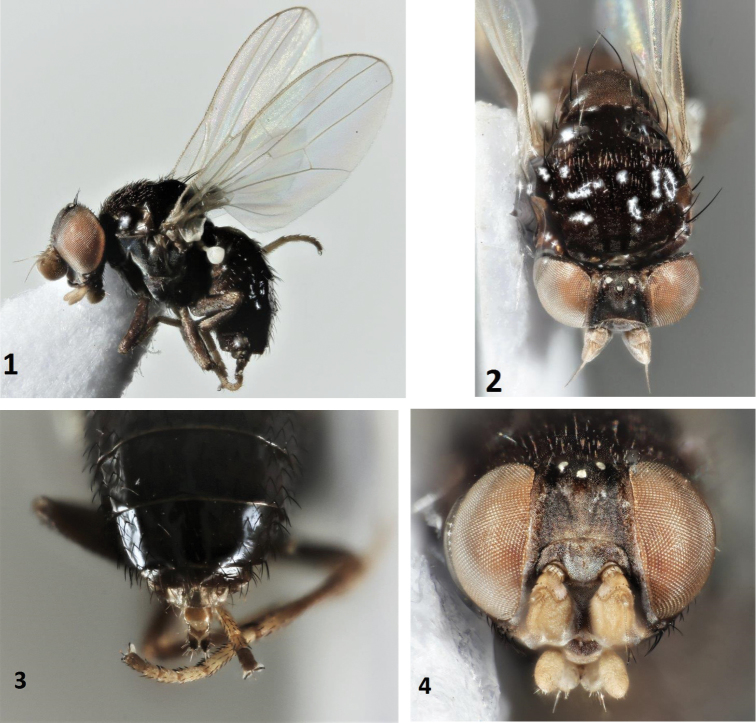
*Leucopis* sp. n., female; **1** habitus, lateral **2** head and thorax, dorsal **3** abdomen dorsal **4** head anterior.

**Distribution**: Turkey.

***Leucopis* sp.**

**Material examined**: 1♂, 13 km NE of Muğla, pinewood + pasture, 1100–1300 m, 37°15'N, 28°30'E, 2–3.v.2016.

**Remarks**. A single male specimen of *Leucopis* could not be identified. It is probably a variant of one of the commoner species as it shows no differentiating external characters but only small differences in the shape of the aedeagus. Without more material it is not possible to come to any definitive conclusion on the taxonomic status of this specimen.

***Leucopomyia* Malloch, 1921**

***Leucopomyiapalliditarsis* (Rondani, 1875)**

Kaydan et al. 2006: 333 (as *Leucopomyiaalticeps* (Czerny, 1936))

**Distribution**: from Iberian Peninsula through Central Europe to Russia and Middle Asian states.

***Leucopomyiasilesiaca* (Egger, 1862)**

Ülgentürk 1999: 76, 2001: 371; Kaydan et al. 2006: 333

**Distribution**: From Britain through Central Europe, Russia, Ukraine, to Middle Asian states.

***Neoleucopis* Malloch, 1921**

***Neoleucopisatratula* (Ratzeburg, 1844)**

Eichhorn 1968: 210; McAlpine 1971: 1868; Tanasijtshuk 1986: 173

**Distribution**: From Britain through Central Europe to the Balkan states and Turkey. Introduced into North America, Hawaii, New Zealand, and Argentina.

***Neoleucopiskartliana* (Tanasijtshuk, 1986)**

Düzgüneş et al. 1982: 92 (as *Leucopiscaucasica* Tanasijtshuk, 1961); Ülgentürk et al. 2013: 533

**Material examined**: 1♀, Akyaka, 30 m, forest, SW, 37°03'16"N, 28°19'35"E, 30.iv.–9.v.2013; 1♂, 13 km NE of Muğla, pinewood + pasture, 1100–1300 m, 37°15'N, 28°30'E, 2–3.v.2016.

**Remarks**. Gaimari et al. (2007) provided a detailed redescription with biological notes on this species, studied in Greece, and speculated that it ought to occur in Turkey, evidently unaware that it had been already recorded from there. More information on the biology of this species in Turkey was given by Ülgentürk et al. (2013).

**Distribution**: Georgia, Italy, Greece, Turkey.

***Neoleucopisobscura* (Haliday, 1833)**

Eichhorn 1968: 210; McAlpine 1971: 1862; Tanasijtshuk 1986: 169

**Distribution**: North and Central Europe to the Balkan states and Turkey. Introduced into eastern and western North America.

***Neoleucopistapiae* (Blanchard, 1964)**

McAlpine 1971: 1866

**Distribution**: Europe, from Britain south to Gibraltar and west to western Russia. Introduced to North and South America and New Zealand.

## Conclusions

Many scientists consider Anatolia to have been an important Pleistocene glacial refugium, which together with the heterogeneous topography and geographical position of Anatolia at the junction of three biodiversity hotpots, the Caucasus, Irano-Anatolian, and Mediterranean (Gür 2016), may have contributed to a very high animal diversity. This, alongside an insufficient level of faunistic research, may explain the recent increase in the number of known Chamaemyiidae from Turkey.

Turkey may have one of the most diverse faunas of Chamaemyiidae in the Southern Palaearctic. We list 40 species in five genera including seven new records and one undescribed new species. Notwithstanding this list, we think the fauna still remains poorly known. There are several species present in adjacent countries that have not yet been found in Turkey, a country that offers a very diverse topography and plant life. Sampling in as many diverse habitats as possible, in different seasons, and rearing silver flies from populations of their hosts will yield interesting results, thus adding to the knowledge of the biology and ecology of this family.
